# Supplementary figures and images for: Scenarios of availability of water due to overexploitation of the aquifer in the basin of Laguna de Santiaguillo, Durango, Mexico
Source: PeerJ. 2019 Jul 15;7:e6814. doi: 10.7717/peerj.6814 (PMC6637934; doi:10.7717/peerj.6814)

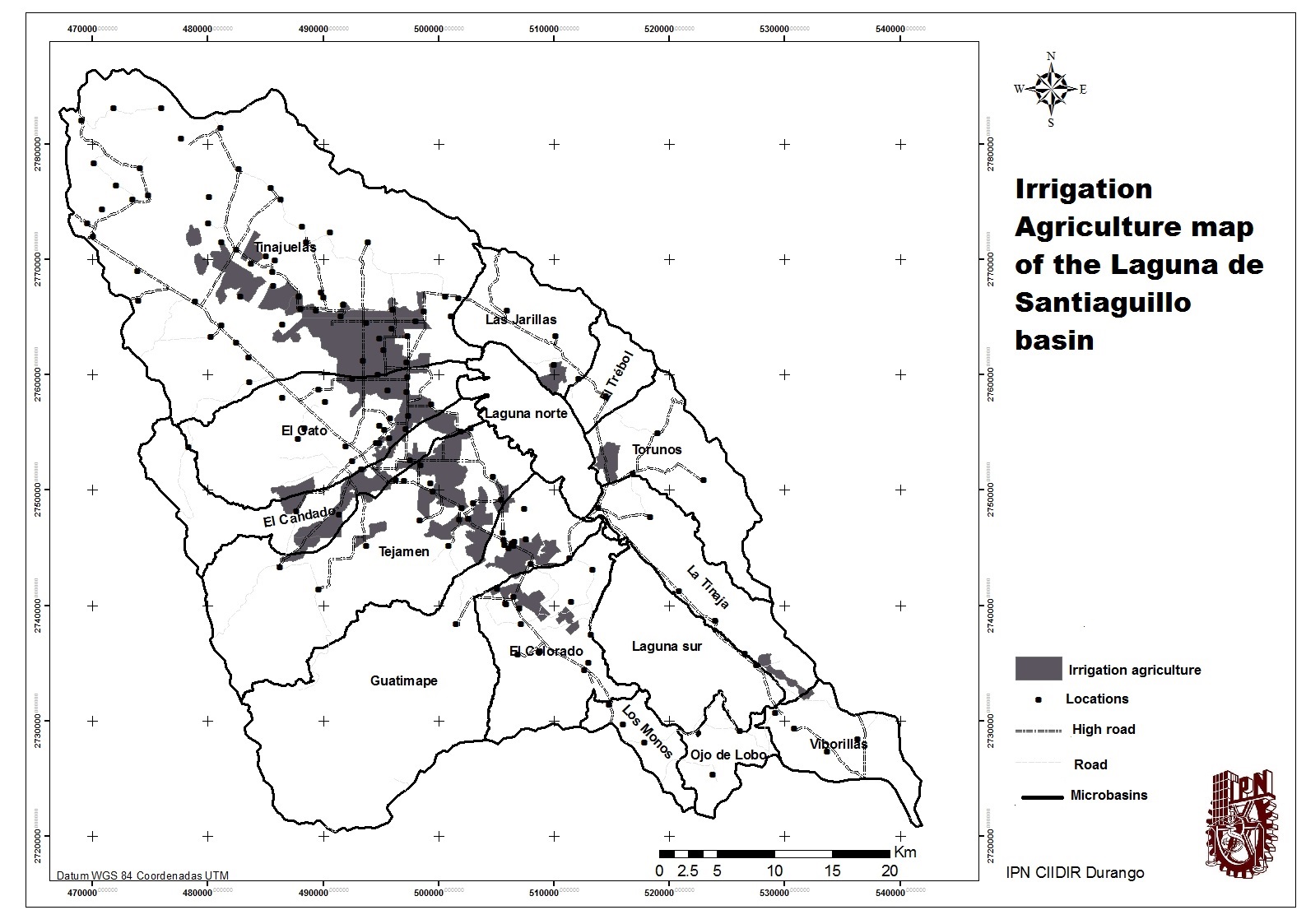

Supplement: Supplemental Information 2 [file peerj-07-6814-s002.jpg]

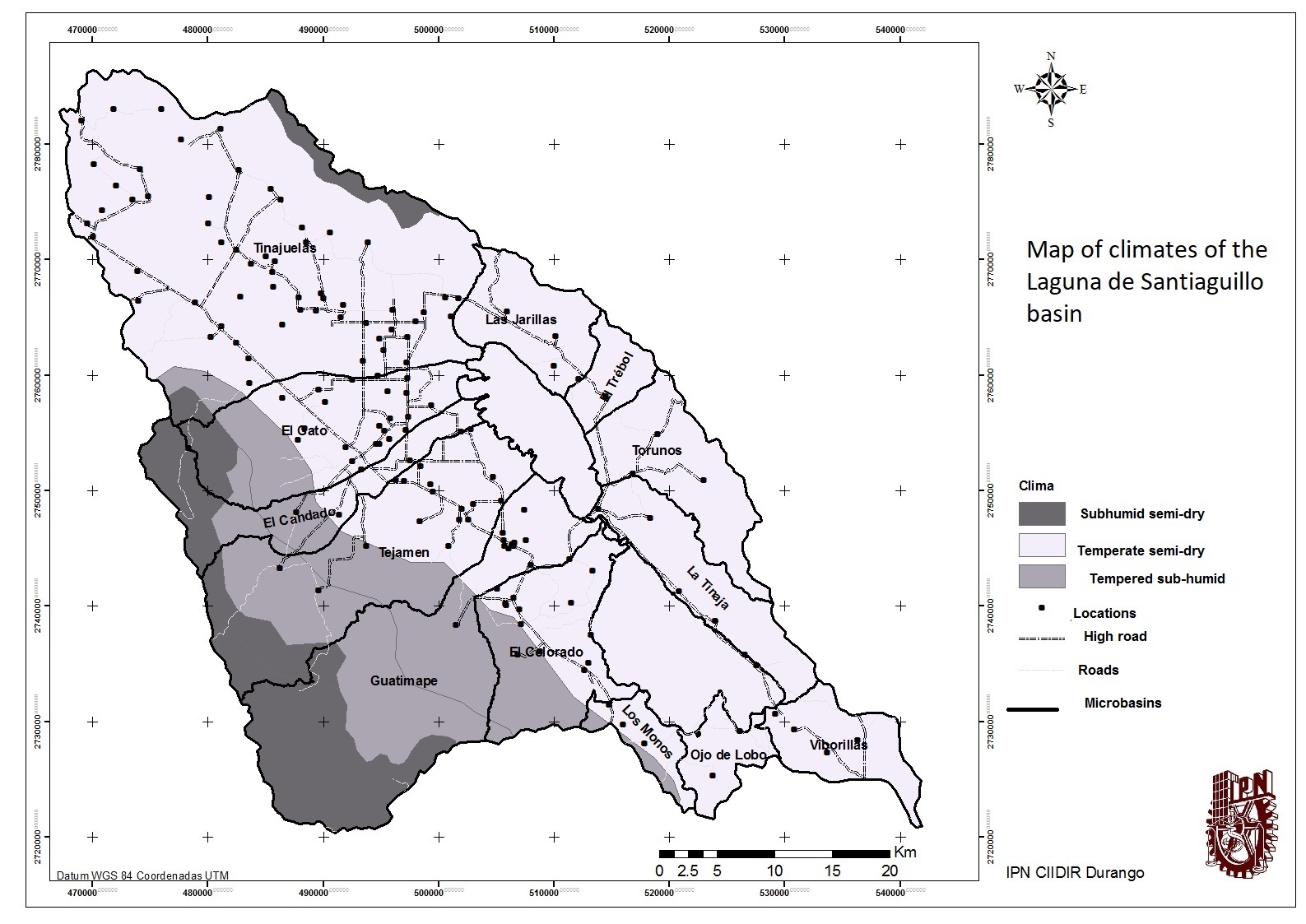

Supplement: Supplemental Information 3 [file peerj-07-6814-s003.jpg]

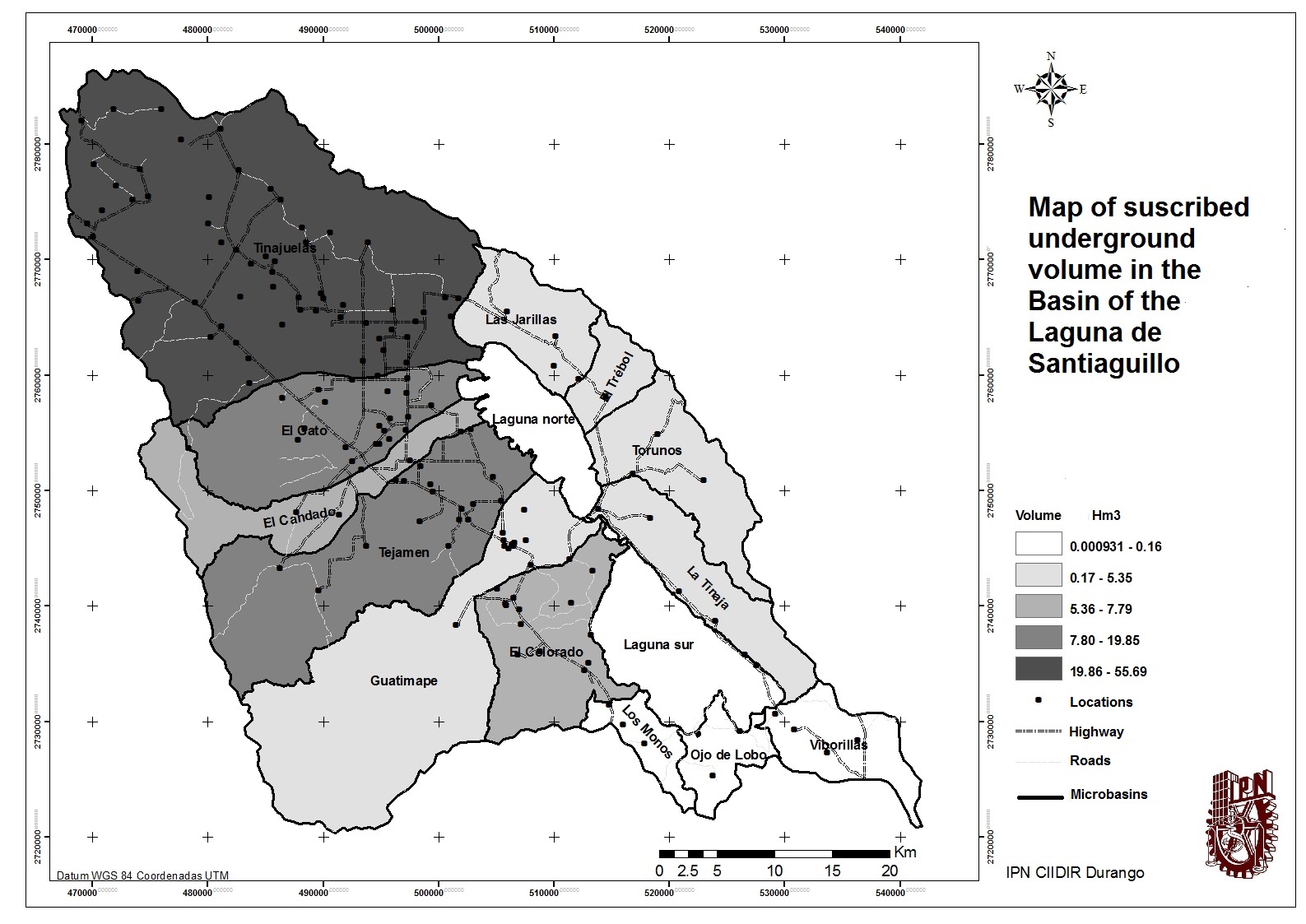

Supplement: Supplemental Information 4 [file peerj-07-6814-s004.jpg]

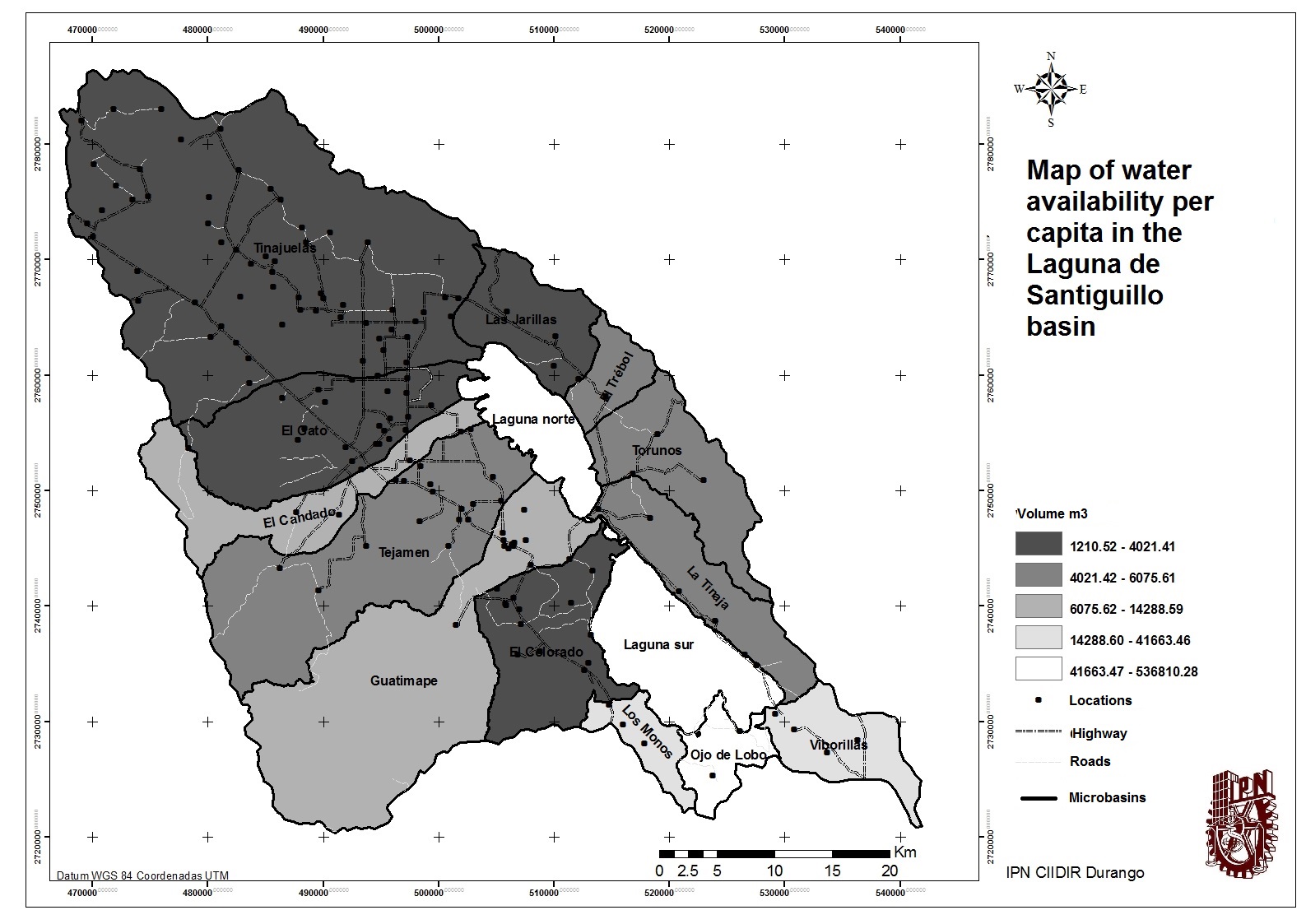

Supplement: Supplemental Information 5 [file peerj-07-6814-s005.jpg]

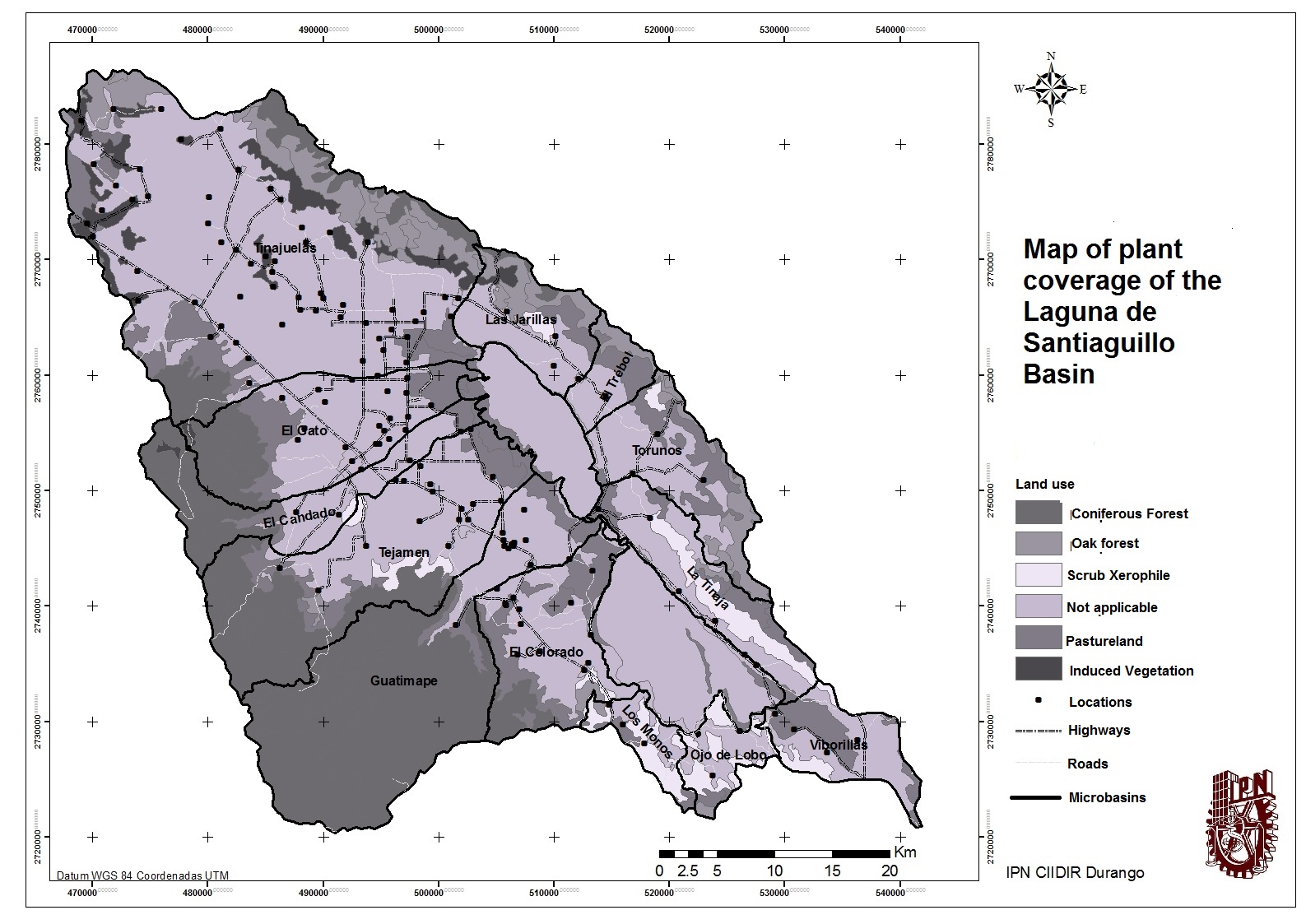

Supplement: Supplemental Information 6 [file peerj-07-6814-s006.jpg]
